# Effects of a Passive Back-Support Exoskeleton on Knee Joint Loading during Simulated Static Sorting and Dynamic Lifting Tasks

**DOI:** 10.3390/ijerph19169965

**Published:** 2022-08-12

**Authors:** Mona Bär, Tessy Luger, Robert Seibt, Julia Gabriel, Monika A. Rieger, Benjamin Steinhilber

**Affiliations:** Institute of Occupational and Social Medicine and Health Services Research, University Hospital of Tübingen, 72074 Tübingen, Germany

**Keywords:** knee force, tibiofemoral force, side effects, assistive device, asymmetric lifting, load shift, forward bent posture

## Abstract

Due to the load shifting mechanism of many back-support exoskeletons (BSEs), this study evaluated possible side effects of using a BSE on knee joint loading. Twenty-nine subjects (25.9 (±4.4) years, 179.0 (±6.5) cm; 73.6 (±9.4) kg) performed simulated static sorting and dynamic lifting tasks, including stoop and squat styles and different trunk rotation postures. Ground reaction force, body posture and the force between the chest and the BSE’s contact interface were recorded using a force plate, two-dimensional gravimetric position sensors, and a built-in force sensor of the BSE, respectively. Using these parameters and the subject’s anthropometry, median and 90th percentile horizontal (HOR_50_, HOR_90_) and vertical (VERT_50_, VERT_90_) tibiofemoral forces were calculated via a self-developed inverse quasi-static biomechanical model. BSE use had a variable effect on HOR_50_ dependent on the working task and body posture. Generally, VERT_50_ increased without significant interaction effects with posture or task. HOR_90_ and VERT_90_ were not affected by using the BSE. In conclusion, utilizing the investigated exoskeleton is likely to induce side effects in terms of changed knee joint loading. This may depend on the applied working task and the user’s body posture. The role of these changes in the context of a negative contribution to work-related cumulative knee exposures should be addressed by future research.

## 1. Introduction

Musculoskeletal disorders (MSD), especially in the back, remain the most common health problem affecting workers in the European Union [1] and the United States [2]. Twelve-month prevalence rates of 58% for the occurrence of general MSD [1] and of 25–43% for the back area have been reported [1,2]. Musculoskeletal back pain has been found to be strongly associated to physical risk factors, especially heavy lifting in the workplace and cumulative low back load. Therefore, intervention strategies for physically demanding work incorporating these risk factors need to remain a focus [2,3].

To support workers in their daily work routines, the use of exoskeletons has become a focus area. “Exoskeletons are assistive systems worn on the body that act mechanically on the body. In an occupational context, they aim to support functions of the skeletal and locomotor system during physical work” [4] (p. 3), by transferring forces from exposed body regions to other body sites [5]. Currently, one of the main debates about the use of exoskeletons is whether or not they are effective in preventing work-related MSD [4].

Recently, a growing number of studies have focused on biomechanical, physiological, and subjective stress and strain parameters for determining the impact of using exoskeletons on the musculoskeletal system during occupational tasks [6]. Passive back-support exoskeletons (BSEs) were shown to potentially reduce physical strain in the supported body area in experiments including dynamic tasks such as lifting [7,8,9,10,11] and in tasks with a static forward bent posture [12,13,14,15,16]. Describing the short-term influence of exoskeletons on physical stress and strain parameters in the supported body area is one important step toward identifying potential strategies for relieving these specific musculoskeletal structures in the wearer.

However, the nature of many exoskeletons is shifting the mechanical load from one area to a different area or areas of the body [5], which raises concerns about excessive biomechanical stresses on these other areas [4,17]. In this context, potential side effects of using BSEs have been examined using parameters such as muscle activity and perceived discomfort outside the target region [10,11,12,18,19,20,21,22,23]. With respect to side effects, findings for strain parameters in the legs, i.e., mean muscle activity and perceived discomfort have been inconsistent. Some studies report decreases [12,20,21,22], others report increases [10,11,18,19,20], or no statistically significant changes [10,11,12,18,19,23,24]. The ambiguous findings of the available studies and the fact that only few focused extensively on possible side effects in the leg region of using a BSE show that it is unclear whether and how using a BSE affects the musculoskeletal system of the lower limb [6].

To ensure the safe application of BSEs, including the aim of promoting workers’ health in physically demanding work, it is imperative to further investigate potential side effects (i.e., potential adverse consequences) of their use. An evaluation of biomechanical joint loading might also give more insight into load transfers or load shifts to other (i.e., non-supported or non-targeted) body areas caused by using a BSE [6,25]. Although the back, shoulder, and neck are much more commonly affected by MSD than the knee, Govaerts et al. (2021) reported a 33% overall prevalence of work-related MSD in the knee among industrial workers [26]. Moreover, there is reasonable evidence that knee disorders are related to physical work exposures partially similar to those reported for back pain, including awkward postures, lifting, and task repetition [1,27]. To the authors’ knowledge, so far there has been no published study focusing on the mechanical loading of lower limb joints, particularly the knees, when using a BSE. Hence, the aim of this study was to evaluate the horizontal (anteroposterior) and vertical forces acting on the tibiofemoral joints when using a BSE (Laevo^®^, Delft, The Netherlands) during simulated industrial work tasks. For this purpose, a self-developed two-dimensional inverse quasi-static biomechanical model was used. We hypothesized that the horizontal and vertical median and 90th percentile tibiofemoral forces increase when using the Laevo^®^ exoskeleton.

## 2. Materials and Methods

### 2.1. Sample Size and Study Design

This manuscript comprises one section of a broader, exploratory laboratory experiment, evaluating the effects of the Laevo^®^ V2.56 exoskeleton on physiological and biomechanical parameters using a within-subject-design [28] (registered at ClinicalTrials.gov, NCT03725982). A Single Williams Latin Square design [29] for six conditions ((1) *Exoskeleton*: Laevo^®^ exoskeleton (*EXO*) vs. *Control*; (2) *Task*: *Static* vs. *Dynamic*; (3) *Lifting style*: *Stoop* vs. *Squat*) was used to determine the sample size of 36 and to randomize the order of the main experimental tasks in this study. In addition, a Double Williams Latin Square design [29] was applied to randomize three *Trunk orientation* conditions for the tasks. The order of randomization resulted from drawing lots.

### 2.2. Participants

Thirty-nine male subjects were recruited to participate in the study, of which three subjects had to be excluded due to time restrictions (N = 1) or not meeting the BMI criterion (N = 2). Thirty-six healthy males completed the experiment, of which data from 29 subjects (mean age 25.9 (±4.4) years, mean body height 179.0 (±6.5) cm, mean body weight 73.6 ± 9.4 kg) were used for the outcome measures described here. The force plate data from seven subjects could not be used due to technical issues. Inclusion criteria were: male gender, age (18–40 years), BMI (18.5–30 kg/m^2^), and absence of any acute or cardiovascular diseases, physical disabilities, systemic diseases, or neurological impairments that would not allow subjects to perform the tasks or wear the exoskeleton. BMI was calculated by measuring body height and weight, while the other inclusion criteria were assessed according to subjects’ self-report. These restrictions in our study sample were chosen to avoid possible moderating influences of sex/gender, age, or even body composition, which have not previously been studied. Furthermore, male subjects were chosen due to the domination of males in the manufacturing industries. The Laevo^®^ exoskeleton is only adjustable to a restricted extent and might therefore not fit to all body dimensions (e.g., female body composition, BMI > 30 kg/m^2^) We chose a rather young age group to ensure that all subjects were able to perform the tasks without an early onset of fatigue.

The study was designed according to the Declaration of Helsinki and approved by the Ethics Committee of the University and University Hospital of Tübingen (617/2018BO2).

### 2.3. Exoskeleton

We evaluated the passive exoskeleton Laevo^®^ (V2.56, Laevo B.V., Delft, The Netherlands; 2.8 kg), which supports the back during work tasks such as lifting a load and tasks requiring forward bending postures. Torque generation is provided by two two-dimensional joints (“smart joints”) with gas pressure springs that are attached to a hip belt located close to the pivot point of the hip joints. Two rigid bars connect the joints to a chest pad placed over the upper part of the sternum and to two leg pads placed over the thighs. The smart joints can be turned on and off, and the joint flexion angle at which the support should begin can be set (range 0–45°, increments of 5°). The exoskeleton was adjusted to fit the subject’s physique in two ways: First, by varying the size of the exchangeable rigid bars connecting the chest pad and the smart joints resulting in a chest-to-smart joint distance of 405 mm (S-size) or 435 mm (L-size). Secondly, by adjusting the smart joint support angle to avoid contact forces while standing upright (depending on the subject’s torso composition). The force was measured and controlled using an integrated force sensor in the chest pad (38 × 10 mm; Type KM38-1kN, ME-Messsysteme GmbH, Henningsdorf, Germany). The leg-pad-to-smart joint distance could not be adjusted and was always 200 mm.

### 2.4. Experimental Procedure and Tasks

**Figure 1:**
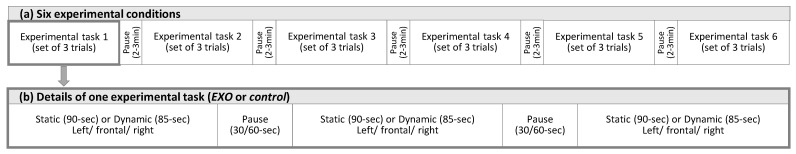
(**a**) shows the sequence of the six experimental conditions; two static and four dynamic: *Static-EXO; Static-Control; Dynamic-EXO-Stoop; Dynamic-Control-Stoop; Dynamic-EXO-Squat; Dynamic-Control-Squat.* The six conditions were performed in randomized order, and each was performed in a set of three *Trunk orientations.* Each set of static sorting tasks lasted 330 s, and each set of dynamic lifting tasks lasted 375 s. (**b**) shows one set of one experimental task. *Trunk orientations* (*left/frontal/right*) were performed in randomized order. Figure modified after Bär et al. (2022) [16].

A 1.5-h visit to our laboratory was mandatory 1–5 days prior to participating in the experiment. This visit included information about the study procedure and signing an informed consent form. Inclusion and exclusion criteria were clarified, anthropometric measurements were collected, and subjects were familiarized with the exoskeleton and tasks. On the day of the experiment, which lasted 4 h, the subject was prepared with the measurement equipment required for the outcome measures and performed a series of experimental tasks [11,19,24]. This manuscript considers six experimental task conditions (*Static-EXO; Static-Control; Dynamic-EXO-Stoop; Dynamic-Control-Stoop; Dynamic-EXO-Squat; Dynamic-Control-Squat*; cf. Figure 1) and focusses on the outcome measures related to knee forces (i.e., tibiofemoral forces).

**Figure 2:**
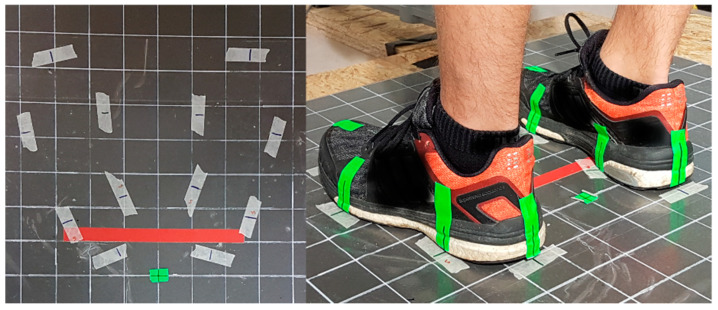
Force plate prepared with a coordinate system and with the individually pre-adjusted and pre-marked foot positions for the different tasks (static and dynamic). Marked landmarks were the heel in line with the Achilles tendon, medial and lateral malleolus, medial and lateral sesamoid, and the forefoot. Tape was placed on the subjects’ shoes and on the force plate, and a connecting line was drawn between each pair of foot-to-floor tape markings considering the above outlined landmark positions. The malleolus markers were later used to determine the x and y coordinates of the ankle joint centers for both feet; by calculating the midpoints of the lateral and the medial malleoli.

The experimental tasks were performed while standing on a force plate in front of a table that was adjustable according to the subject’s height. The feet position was defined prior to the experiment and kept constant during each task by using markings on the force plate (Figure 2). The feet position was defined while the subjects were instructed to stand comfortably upright with their feet positioned evenly and facing straight ahead. The distance to and height of the table was adjusted to allow the subject to perform the sorting or lifting task in the required body postures (explanation below). The six experimental conditions were performed in sets of three trials, with each trial performed in one of the three trunk orientations. Therefore, the sorting or the lifting box was placed to the front, in a 45° rotation to the left, or in a 45° rotation to the right from the sagittal plane. Reported results in the frontal direction include both knees when the work tasks were performed without trunk rotation. The reported ipsilateral results refer to both trunk orientations (left and right), including the knee belonging to the body side that coincides with the direction of trunk orientation. The reported contralateral results refer to both trunk orientations (left and right), including the knee belonging to the side of the body opposite to the direction of trunk orientation.

**Figure 3:**
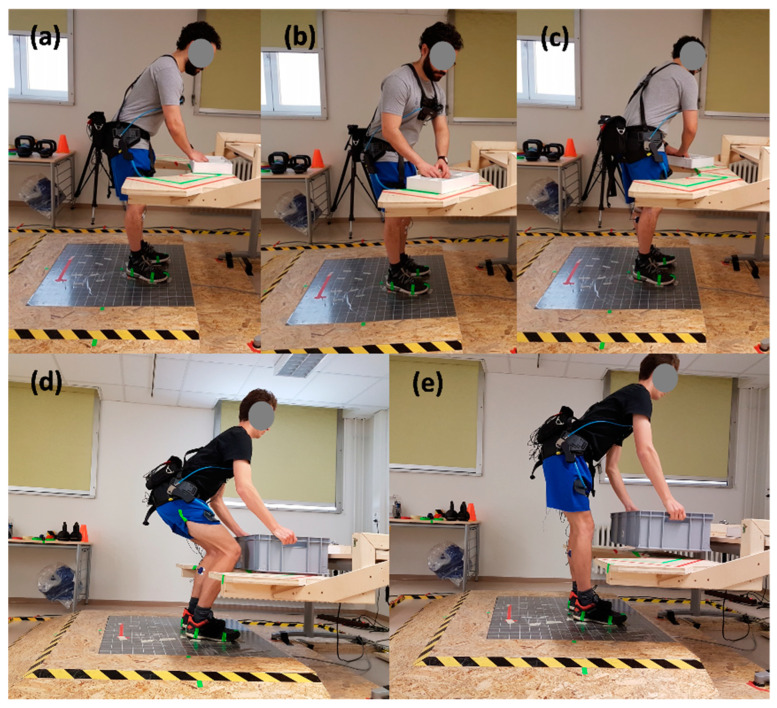
Subjects performing the experimental work tasks using the exoskeleton. (**a**–**c**) [16] show the static sorting task in three *Trunk orientation* conditions: (**a**) frontal, (**b**) right orientation, (**c**) left orientation. (**d**,**e**) show the dynamic lifting task to the front, performing (**d**) the squat style and (**e**) the stoop style.

The simulated static work task included sorting screws and pins while keeping the trunk in a 40° forward bent posture in the sagittal plane, following the tangent line of a two-dimensional gravimetric position sensor (PS12-II; Thumedi GmbH & Co. KG, Thum, Germany) that was placed on the skin over the spinous process of the 10th thoracic vertebrae (T10). The examiner monitored the signal on a screen. Additionally, the subjects were instructed to almost completely extend but never overstretch their knees (stoop knee posture) and to keep their feet in the pre-marked position. The height of the table was adjusted and the y-position of the feet was set while the subjects remained in the forward bent posture, comfortably reaching the sorting material with their hands while their elbows were flexed at approximately 135°. The sorting task lasted 90 s without moving the feet, legs or trunk, and was performed in the two following conditions: with or without the exoskeleton (*Static-EXO* vs. *Static-Control*). The subjects rested for 30 s between each trunk orientation and for 120 s after each static experimental condition. (Figure 3a–c).

The simulated dynamic work task included lifting and lowering an 11.6 kg load (i.e., a 10 kg load placed into a 1.6-kg box [W × D × H of 60 × 40 × 22 cm] with handles on both sides [19 cm]). The pre-defined body posture for adjusting the table included bending the upper body at a 70° flexion-angle in the sagittal plane, controlled similarly to the static task, with the legs almost completely extended but not overstretched. The upper arms hung perpendicular to the platform with an elbow flexion of approximately 160° while holding the handles of the box. Each dynamic experimental condition consisted of two sets of five consecutive lifts, keeping a pace of 5 s per lift, timed by an acoustic signal. The subjects rested for 35 s between both sets. Each lifting repetition included the following movements: (1) starting in an upright standing position, bending the trunk forward and picking up the load; (2) resuming the upright position while holding the load close to the body in front of the pelvis with flexed elbows; (3) lowering the load by bending the trunk forward and returning the load to its original position; (4) resuming the initial upright standing position without the load. The lifting task was performed in the following four conditions: with or without the exoskeleton, and holding the knees almost extended (stoop style) or bending the knees (squat style) while lifting (*Dynamic-EXO-Stoop* vs. *Dynamic-Control-Stoop* vs. *Dynamic-EXO-Squat* vs. *Dynamic-Control-Squat*). The subjects rested for 60 s between each trunk orientation and for 180 s after each dynamic experimental condition (Figure 3d,e).

All tasks were approved for their work-related relevance by consulting seven industrial companies who were already testing or had interest in testing BSEs in their companies. The applied tasks and their executions, i.e., body postures, lifting frequency, working height, have best represented the real work situations of these consulted companies.

### 2.5. Measurement and Data Analysis

The outcome parameters to assess the forces acting on the tibiofemoral joints during the dynamic work task were 50th and 90th percentile horizontal (anteroposterior) forces (HOR_50_, HOR_90_), and 50th and 90th percentile vertical forces (VERT_50_, VERT_90_). They were considered as median and peak knee loads during the lifting tasks. During the static work task, only HOR_50_ and VERT_50_ were estimated, since the static body posture over the 90-s sorting task period would induce 90th percentile forces which do not differ much from the median forces. To estimate the forces acting on the knee joints, an inverse quasi-static model was developed, since no established model incorporating the Laevo^®^ exoskeleton that could be applied was available (see Appendix A for a detailed explanation of the model). Quasi-static models have been used previously to detect the risk of injury in industrial workers [30]. For the model, subjects’ anthropometrics, including body height, segment lengths, and segment weights [31,32,33], distances between the devices’ contact points, lower limb posture, ground reaction forces below the feet, and the force between the chest and the exoskeleton’s contact surface, were recorded.

To measure the lower limb posture, we used gravimetric inclination sensors connected to a sampling and storage device (PS12-II with 2.5D-gravimetrical sensors; THUMEDI GmbH & Co. KG, resolution 0.1° and 125 ms in time; maximum static error 0.5°; maximum repetition error 0.2°) attached to the skin over the anterior tibia and femur using double-sided adhesive tape (25 × 20 mm, 3M transparent Medical Standard, Top Secret^®^, Gesellschaft für Haarästhetik mbH, Fürth, Germany). The measurement system continuously recorded the anteroposterior and lateral inclination angles respective to the gravitational axis. Possible angular offsets caused by individual placement of the sensors at the tibia and femur were neutralized using the measurement values of a 5-s upright standing period recorded prior to the experiment.

Ground reaction forces were continuously recorded using a three-dimensional force plate that was linked to a signal conditioner and digitizer (FP9090-15-1000; Analog and Digital Amplifier AM6800; resulting resolution 0.5 N and 125 ms in time; overall maximum error 6 N; Bertec Corporation, Columbus, OH, USA). The resulting digital force signals were continuously recorded by self-developed software (University Hospital Tübingen) using the Bertec “Device interface Library for NET”, which allows recoded data to be synchronized with data captured by the inclination sensors placed on the lower limbs.

The force plate’s platform was prepared with a coordinate system to determine the subject’s standing position, which was kept constant for the static and dynamic tasks (see 2.4 Experimental procedure and tasks; Figure 2). The points forming the tangent between the lateral and medial malleoli were marked on the coordinate system and used for further calculations. Prior to each measurement session, a self-calibration procedure was executed to remove possible offsets, for example, caused by temperature variations. The position measurement accuracy was regularly checked by placing a 2 kg weight on five predefined locations on the force plate (at the center and close to the four corners); accuracy was accepted with measured location errors < 10 mm.

The support moment of the exoskeleton was estimated by measuring the contact force between the Laevo^®^ exoskeleton and the chest using a Ø38 mm × 10 mm thick force sensor (Type KM38-1kN, ME-Meßsysteme GmbH, Henningsdorf, Germany; resolution 0.1 N; maximum error 1% = 10 N, shown to be <2.5 N in this study setting) that was manually integrated in the chest pad of the exoskeleton and connected to the previously described sampling and storage device (PS12-II, 24 Bit physical resolution, 4096 Hz sampling rate).

Several of the subjects’ anthropometrics (i.e., body height, body weight), segment lengths, and distances (i.e., shank and thigh lengths, distances between sesamoid and malleolus) and segment distances of the exoskeleton (i.e., distance between joints and contact points) were included in the model for the force calculations (Cf. Appendix A).

### 2.6. Statistical Aanalysis

The normal distribution of the histograms of the outcome parameters was inspected visually and the absolute *z*-values of the skewness and kurtosis of the data were judged to be valid for statistical evaluation [34]. We used repeated-measures analyses of variance (RM-ANOVA) with fixed factors *Exoskeleton (E), Trunk orientation (TO)* and *(E × TO)* to analyze differences between the experimental conditions (1) *Static-EXO* vs. *Static-Control* for the outcome parameters HOR_50_ and VERT_50_. We used RM-ANOVA with fixed factors *E, TO, Lifting Style (LS), E × TO, E × LF, TO × LS, and E × TO × LS* to analyze differences between the experimental conditions (2) *Dynamic-EXO-Squat* vs. *Dynamic-Control-Squat*, and (3) *Dynamic-EXO-Stoop* vs. *Dynamic-Control-Stoop* for the outcome parameters HOR_50_, HOR_90_, VERT_50_, VERT_90_. However, only the findings including the *Exoskeleton*-condition are presented in the results section. To evaluate the static sorting task, we included the full 90-s periods. To evaluate the dynamic lifting task, we included only the two phases including the weight: (2) resuming the upright position while holding the load and (3) lowering the load by bending the trunk forward and returning the load to its original position. If statistically significant interaction effects occurred, Student’s *t*-tests were used for post-hoc pairwise comparisons. Further interpretations only considered the relevant comparisons (i.e., *EXO* vs. *Control* within each *Trunk orientation*: *ipsilateral*, *frontal*, *contralateral*, within each *Lifting style: Stoop, Squat*, and within the combination of *Trunk orientation* and *Lifting style*). For fixed effects, *F*-values, *p*-values, and effect size partial eta squared ($\eta_{p}^{2}$) were calculated using the *F*-ratios strategy [35], and for the post-hoc pairwise comparison, *T*-value, *p*-value, and effect size Cohen’s *d* were calculated using the pooled standard deviation strategy [36]. In agreement with Cohen [36] and *F*-ratios strategy [35], effect sizes were interpreted as small ($\eta_{p}^{2}$ ≤ 0.02; *d* ≤ 0.2), medium ($\eta_{p}^{2}$ 0.13–0.259; *d* 0.5–0.79), or large ($\eta_{p}^{2}$ ≥ 0.26; *d* ≥ 0.8). For pairwise comparisons, we accepted significance levels of α ≤ 0.05 for fixed effects, and of α ≤ 0.00333 for *E × TO*, α ≤ 0.00833 for *E × LS,* and α ≤ 0.00076 for *E × TO × LS* (Bonferroni correction for 15, 6, and 66 possible comparisons, respectively). JMP^®^ (Version 14.2.0, SAS Inc., Carry, NC, USA) was used for statistical evaluations.

## 3. Results

Median values with corresponding interquartile ranges (IQR) and differences between *EXO* and *Control* are provided in Table 1 for the main comparisons of the static and dynamic work tasks, in Table 2 for the *E × TO* and the *E × LS* comparisons for the static and dynamic work tasks, and in Table 3 for the *E × TO × LS* comparisons of the dynamic work task. The related statistics for the main effects of the *Exoskeleton* condition (*EXO* vs. *Control*) and the interaction effects for *E × TO, E × LS, and E × TO × LS* are provided in Appendix B (Table A5) for all examined work tasks. All relevant pairwise comparisons for variables with significant interaction effects are provided in Appendix B (Table A6) for static and dynamic work tasks.

### 3.1. Static Task

In the static work task, *Exoskeleton* had no significant main effect on HOR_50_. However, there was a significant interaction effect for *E × TO* (*p* < 0.001; $\eta_{p}^{2}$ = 0.496), including a significant pairwise comparison for the *contralateral* side (*p* < 0.001; *d* = −0.912), which showed a reduction when using the *EXO* (−126.4%) (Cf. Appendix B).

The main effect of *Exoskeleton* was significant for VERT_50_ (*p* = 0.011; $\eta_{p}^{2}\;$= 0.209); the acting force increased (1%) when using the *EXO*. There was no significant interaction effect for *E × TO* on VERT_50_ (Cf. Table 1 and Appendix B).

### 3.2. Dynamic Task

Performing the dynamic work task, *Exoskeleton* had a significant main effect on HOR_50_ (*p* = 0.012; $\eta_{p}^{2}=$ 0.205) with significant interaction effects for *E* × *TO* (*p* < 0.001; $\eta_{p}^{2}=0.455$), *E* × *LS* (*p* < 0.001; $\eta_{p}^{2}$ = 0.471), and *E* × *TO* × *LS* (*p* = 0.002; $\eta_{p}^{2}=$ 0.201). Pairwise comparisons for *E* × *TO* were significant only for *contralateral* (*p* < 0.001; *d* = −0.493). Pairwise comparisons for *E* × *LS* was significant only for *Squat* (*p* < 0.001; *d* = −0.261). Pairwise comparisons for *E* × *TO* × *LS* were significant for *E* × *ipsilateral* × *Squat* (*p* < 0.001; *d* = −0.195), for *E* × *frontal* × *Stoop* (*p* < 0.001; *d* = 0.597), for *E* × *contralateral* × *Squat* (*p* < 0.001; *d* = −0.487), and for *E* × *contralateral* × *Stoop* (*p* < 0.001; *d* = −0.717). (Cf. Appendix B) *EXO* decreased HOR_50_ when performing the task in *Squat style* in all directions (−61.1–−19.8%), and increased HOR_50_ when performing the *Stoop style* in *ipsilateral* and *frontal* (+13.2; +20.6%) and decreased HOR_50_ when performing the *Stoop style* in *contralateral* (−85.5%) (Cf. Table 3).

*Exoskeleton* had no significant main effect on HOR_90_. However, there was a significant interaction effect for *E* × *TO* (*p* < 0.001; $\eta_{p}^{2}$ = 0.292) and *E* × *LS* (*p* = 0.006; $\eta_{p}^{2}$ = 0.236), but without reaching statistical significance in the relevant pairwise comparisons and without interaction effects for the threefold interaction *E* × *TO* × *LS* (Cf. Appendix B).

*Exoskeleton* had a statistically significant main effect on VERT_50_ (*p* < 0.001; $\eta_{p}^{2}$ = 0.376) without any significant interaction effects (Cf. Appendix B). VERT_50_ slightly increased when using the *EXO* (≤ 7%) (Cf. Table 3).

Using the *EXO* had no significant effect on VERT_90_ (Cf. Appendix B).

### 3.3. Support Moment

Descriptive information about the 50th and 90th percentile support moment of the exoskeleton while performing the work tasks is provided in Table 4.

## 4. Discussion

Numerous studies have evaluated the use of occupational BSEs on short-term changes in physical stress and strain parameters in the body region supported by the exoskeleton. Only a few studies also investigated potential side effects of using occupational BSEs [6]. Therefore, the present study includes the evaluation of biomechanical knee joint loading when using a BSE. Using the Laevo^®^ exoskeleton had a variable influence on the anteroposterior acting horizontal forces, which seems to depend on the work task execution (e.g., lifting style) or posture (e.g., trunk orientation). Yet it remains unclear, whether the occurring changes are relevant in terms of knee joint health. Furthermore, vertical acting forces slightly increased due to the exoskeleton’s weight itself.

When performing the static sorting task in a forward bent static upper body posture with lateral trunk orientation, the ipsilateral knee was heavily loaded and the contralateral knee was almost unloaded. With respect to the horizontally acting forces on the femoral part of the knee joint, only the contralateral knee was significantly influenced by wearing the *EXO*. Without the *EXO*, the force mainly acted in anterior direction (*Static*-*Control-contralateral:* 20.7 ± 23.4 N), and with the *EXO* in a more posterior direction (*Static-EXO-contralateral*: −5.5 ± 48.3 N). The mechanical principle of transmitting load from the back to the leg pads via smart joints induced a translation force directed backwards onto the thighs [12], causing a posteriorly directed knee force.

Performing the dynamic work task using the *EXO* had an overall influence on HOR_50_. The major effect for work direction was observed on the *contralateral* side, reducing the anteriorly directed HOR_50_ for both *Lifting styles* (*Squat-contralateral*: −61.1%; *Stoop-contralateral*: −85.5%), while still being anteriorly directed (*Squat-EXO-contralateral:* 19.6 ± 69.5 N; *Stoop-EXO-contralateral*: 4.3 ± 58.6 N). Within the *E × LS* interaction, only the *Squat style* had a significant effect, reducing the anteriorly directed HOR_50_ even during frontally directed work and on the ipsilateral side during lateral work. In contrast, HOR_50_ tended to increase when performing *Stoop style* lifts during frontally directed work and during laterally directed work on the ipsilateral side, similar to our findings for the static work task. Both tasks were performed while maintaining almost extended knee postures compared to the *Squat style* (median flexion in *Static*: 19.7°; median and peak flexion in *Dynamic_Stoop*: 17.7°, 31.8°; median and peak flexion in *Dynamic_Squat:* 39.2°, 73.7° (0° flexion referring to fully extended knees)). Therefore, it is most likely that the effects of using the Laevo^®^ exoskeleton on horizontal knee forces depend on the wearer’s body posture (i.e., the knee flexion angle).

Shear force magnitudes and directions (anteriorly vs. posteriorly directed) have been shown to vary depending on knee flexion angles in isokinetic knee extension tasks [37,38]. As described in two associated publications [11,16], using the *EXO* led to more flexed knee joints in the static and dynamic tasks. The changes were most prominent in those tasks that included stoop postures (*Static*; *Dynamic_Stoop*), particularly *contralateral* (+95% in *Static*; +78.8% in *Dynamic_Stoop*) where we also detected most HOR_50_ changes when using the *EXO*. Accompanying the knee joint angle changes, the hip joints were also more flexed by the subjects when using the *EXO*, particularly in those tasks including stoop postures and observing the *contralateral* side [11,16]. Further, the support moment of the Laevo^®^ has been shown to depend strongly on the flexion angle of the smart joints which are located close by the hip pivot points [13]. Therefore, the leg pad pressure acting on the thighs must depend on the hip flexion angle, further influencing the horizontally acting knee forces.

Using the *EXO* had no effect on HOR_90_. It is likely that the EXO does not substantially alter peak horizontal forces when lifting and lowering a load in *Stoop* and *Squat Lifting styles*. Therefore, the Laevo^®^ presumably does not induce high peak horizontal loads on the knee joint. However, substantial time spent on knee straining work tasks, including those tasks without substantial force peaks (e.g., holding a posture), has been reported to be an important risk factor for musculoskeletal disorders in the knee [39,40]. Whether exoskeleton-induced changes in HOR_50_ can increase the risk for MSD is beyond the findings of the present study.

To our knowledge, there is no evidence on quantitatively reported knee forces in industrial work tasks and on potential changes induced by workplace interventions. Further, existing evaluations of knee forces, e.g., during daily activities, have been evaluated using different methods (i.e., in vivo measurements via telemetry, different biomechanical models) [41], which makes comparisons difficult. However, in previous studies, anteriorly directed knee forces evaluated during activities of daily living (i.e., walking, ascending and descending stairs, rising from or sitting down in a chair, single or two-legged stance) were reported to range from 0.04–1.6 times body weight (×BW) (peak) [37,42,43,44,45,46] and 0.09–0.18 × BW (mean) [43], and during squatting from 0.11–0.15 × BW (peak) and 0.02 × BW (mean) [42,43]. Posteriorly directed horizontal forces were reported to range from 0.23–1.7 × BW (peak) and 0.12–0.34 × BW (mean) [37,43,44] during daily activities, and from 0.2–3.6 × BW (peak) [37,47] during squatting. Neglecting bias due to insufficient comparability between methods and roughly approximating our data into a multiple body weight (×BW) metric (by dividing each measured force value [N] by the mean body weight of all included subjects (722.02 N)), we obtained the following forces when using the *EXO*: Anteriorly directed horizontal knee forces of ≤0.12 × BW (median) for static work tasks and ≤0.20 × BW (median), and ≤0.67 × BW (peak) for dynamic work tasks. Posteriorly directed horizontal knee forces of <0.01 × BW (median) only for the static task. This is within the force ranges reported for the common activities of daily living, although we included straining postures and additional loads. Therefore, it is possible that using the Laevo^®^ does not exert horizontal forces on the knee joints exceeding typical loads. However, the risk of developing degenerative MSD, such as osteoarthritis in the knee joint, has been shown to be related to cumulative loading over prolonged durations [40,48,49,50]. In Germany, osteoarthritis of the knee and meniscal lesions are listed as occupational diseases for which cumulative knee exposure is an important factor for their recognition [51]. In this context, future research should address a possible negative contribution of BSE use on cumulative loading of the knee joint.

Using the *EXO* had medium to large significant effects on VERT_50_. The force increased in the static work task by 0.9–13.7% (8.2–74.2 N) and up to 7.0% (58.1N) in the dynamic work task, without being influenced by *Trunk orientation* or *Lifting style. EXO* had no effect on VERT_90_. It can be assumed that the increases in VERT_50_ were mainly caused by the exoskeleton’s own weight (39 N; including the inbuilt force sensor), but probably not by any additional load transfer from the back to the legs.

Vertical acting knee forces have been reported to range between 1.0–10.0 × BW (peak) for activities of daily living [37,44,46,52,53,54,55], and to range between 0.3–5.6 × BW (peak) for squat tasks [37,47,54,55,56]. In the present study, when approximating the data into ×BW metrics, the vertical forces with using the *EXO* resulted in 0.48–1.25 × BW (median) for the static task, with 0.54–1.58 × BW (median), and 1.2–2.57 × BW (peak) for the dynamic work tasks. Similar to HOR, this lies within the range of the reported vertical forces when neglecting bias due to insufficient comparability between methods. However, in terms of cumulative knee loading as a risk factor for MSD of the knee, the weight of a BSE may represent a relevant additional load on the musculoskeletal system of the lower limbs. While the weight with 2.8 kg of the Laevo^®^ exoskeleton is rather light, other commercially available BSEs weigh up to 7 kg [57].

Until now, mainly muscle activity has been observed to estimate possible side effects of using BSEs [6]. The loading of the knee joints is highly influenced by forces exerted by the knee extensor and flexor muscles [58]. Therefore, it is likely that changes in occurring knee forces are accompanied by a changed activity of these muscle groups. Previous studies have reported that the knee extensor muscles are only slightly influenced by the use of the Laevo^®^ exoskeleton [9,11,16,18,59]. However, the activity of the gastrocnemius medialis muscle (GM) increased in all dynamic *contralateral* conditions, possibly due to postural changes [11], and the activity of the biceps femoris muscle (BF) decreased across all conditions as reported in two associated publications [11,16], possibly due to the supporting nature of the EXO for hip extension [11,12,16]. It has been reported that antagonistic coactivation of the hamstring is one important knee joint stabilizing factor which also influences forces acting on the knee joint [58]. Both GM and BF contribute to the antagonistic muscle activity with respect to the knee joint during the tasks observed here. Although a BSE may provoke changes in musculoskeletal strain (e.g., muscle activity) by addressing a specific joint (e.g., supporting hip extension), these changes also affect adjacent joints, such as the knee joint. In the present study, changing the activity of BF and GM by using the *EXO* may have produced secondary effects, such as changes of the knee joint forces. This is consistent with the assumptions of Park et al. (2022), who evaluated a BSE during walking and discussed an accompanying reduction in knee flexion torque along with a reduction in hip extension torque due to the hip extension support of the BSE, which may be caused by reduced BF muscle activity [60].

Although this was not explored further in the presented experiment, it is most likely that possible side effects are nearly proportional to the support provided by the device, which is caused by the load-transferring character of the BSE. According to our associated papers, the Laevo^®^ exoskeleton seems to provide a rather low back-relieving effect [11,16]. Consequently, side effects may also occur only slightly. Subsequently, using a BSE that provides a greater amount of support to the user may also cause more accompanying side effects. Further, mechanical differences of different devices may lead to different (side) effects due to the respective mechanical load transfer. For example, Alabdulkarim et al. (2019) compared three exoskeleton designs of upper body support exoskeletons during simulated overhead drilling. The findings demonstrated significant differences between the three exoskeleton designs in muscle activation of the supported area but also different muscle activation in non-target region which can be considered as side effects [61]. Therefore, before implementing a BSE, it is crucial to assess side effects that may occur for each individual device in its current version.

### 4.1. Limitations

Several limitations need to be address for the current study. First, the study population consisted of healthy male subjects aged 19 to 38 years, which does not reflect the general working population, also including female, aging, and physically impaired persons. Therefore, our results cannot be generalized. Second, seven out of originally 36 included subjects had to be excluded for the knee force calculations, resulting in a sample size of 29. Data had to be excluded due to technical issues while synchronizing the data for the first seven subjects. However, the body side that was prepared with the measurement equipment was still counterbalanced (in 14 subjects on the left, in 15 subjects on the right). Third, the Laevo^®^ exoskeleton is only partially adaptable to its wearer’s proportions. The distance between the hip joint and the chest was chosen between two available sizes (S, L), resulting in a lever arm of 405 mm or 435 mm. Only one of the 29 subjects used the S-sized model. The distance between the hip joint and the leg pad was not adjustable for the Laevo^®^, so the leg pads were not always placed exactly as specified by the manufacturer (i.e., lower than instructed for shorter subjects). Variations in exoskeleton placement on the body of the wearer can easily occur when such devices are applied in the field and must be minimized. Similarly, the exoskeleton cannot always be prevented from shifting during all movements. However, in our experiments, the fit of the Laevo^®^ was highly controlled by the examiners. Fourth, all subjects underwent a one-hour familiarization session, which might be too short to fully adapt to a routine exoskeleton use. Fifth, this experiment included three highly controlled simulated work tasks (e.g., on working posture). A real working environment, therefore, was not reflected, and possible variations of the exoskeletons’ effects are not known. Therefore, field studies under randomized, controlled conditions are needed to complement laboratory studies, which only provide initial insights into the acute possible effects of using an exoskeleton. Sixth, this study focused on acute effects of wearing an exoskeleton. Effects induced by regular long-term and full-shift use remain unclear and need to be investigated by long-term studies. Seventh, we used a self-developed biomechanical inverse quasi-static model for calculating moments and forces acting on the joints. The model includes some simplifications that might cause deviations from the actual occurring knee joint forces. Generally, in quasi-static models the dynamic movements are neglected [30] which could have biased the calculated forces in the dynamic lifting task. A comparison of a quasi-static vs. a dynamic model by Hariri et al., (2021) showed an underestimation of peak (19.7%) and cumulative spinal moments (3.6%) when not including the dynamic movements into the model in manual material handling tasks [30]. In particular, the 90th percentile knee forces could have been underestimated in this experiment. Further, only the vertical but not the horizontal components of the ground reaction force were included into the model. Some simplifications regarding the joint mechanics were adopted (i.e., neglecting torsional forces, assuming the pivot point being central and without shifting, treating joints like pure hinge joints, neglecting forces induced by antagonist muscles). The length of some body segments which were used for the model was estimated in relation to the respective body length. Only one leg was prepared with the measurement equipment (i.e., position sensors). Therefore, the force which was generated by the exoskeleton and acted onto the thighs was distributed onto both legs to be 50% loaded each. (Cf. Appendix A for detailed information about the model and its limitations.) Eighth, possible specific effects on the patellofemoral joint could not be assessed by our analysis. Those effects may be induced by the pressure of the exoskeleton’s pad onto the anterior upper leg muscles.

### 4.2. Key Points

- The changes detected for HOR and VERT seem rather small and may not exceed typical ranges. However, it remains unclear what additional effect even small increases in acting knee joint forces have on musculoskeletal knee joint health, considering the contribution of cumulative loads to MSD of the knee.
- This evaluation shows that the side effects of using an exoskeleton depend on the work task executed (i.e., knee and trunk postures). Therefore, the decision to implement a BSE or not needs to depend on the individual work tasks.
- Back-support exoskeletons should be as light as possible, as their own weight seems to directly increase the vertical forces acting on the knee joint.
- Potential side effects, such as changes in knee joint forces, should be considered early in the development of a BSE.

## 5. Conclusions

When developing, evaluating, and applying a BSE, it is crucial to also focus on potential side effects that might occur when using the device during occupational tasks. We found task and posture-related changes in the loading characteristics of the knee joints when using the Laevo^®^ exoskeleton using our biomechanical model. Conclusions regarding the impact on musculoskeletal health risk for the knee would be beyond the present study. However, due to the cumulative nature of MSD, potential negative effects on the knee joints when using BSEs should be considered by future research.

## Figures and Tables

**Table 1:** Median knee force values and corresponding interquartile ranges (IQR), absolute and relative differences showing *EXO* compared to *Control* for static and dynamic work tasks (main interactions).

Work Task	Parameter	Knee Force *Control* [N]		Knee Force *EXO* [N]		Difference (*EXO-Control*)	
		Median	(IQR)	Median	(IQR)	[N]	%
Static	HOR_50_	49.69	(57.28)	46.45	(97.75)	−3.24	−6.5%
	VERT_50_	693.05	(527.15)	700.09	(478.77)	**7.04** **^μ^**	**1.0%**
Dynamic	HOR_50_	52.71	(78.66)	36.56	(96.11)	**−16.15** **^μ^**	**−30.6%**
	HOR_90_	251.91	(279.74)	246.99	(314.22)	−4.92	−2.0%
	VERT_50_	596.91	(376.75)	635.64	(375.45)	**38.74** **^λ^**	**6.5%**
	VERT_90_	1010.14	(604.72)	1041.61	(599.21)	31.47	3.1%

Significant differences are shown in bold (*p*-value α ≤ 0.05). Effect sizes (^λ^ large effect size ($\eta_{p}^{2}$ ≥ 0.26); ^μ^ medium effect size ($\eta_{p}^{2}$ ≥ 0.13)) are shown for the significant differences. Detailed statistics are displayed in Appendix B. N = newton; HOR_50_ = 50th percentile of the horizontal force; HOR_90_ = 90th percentile of the horizontal force; VERT_50_ = 50th percentile of the vertical force; VERT_90_ = 90th percentile of the vertical force.

**Table 2:** Median knee force values and corresponding interquartile ranges (IQR), absolute and relative differences showing *EXO* compared to *Control* for static and dynamic work tasks (two-fold interactions (**a**) *EXO × Trunk orientation* and (**b**) *EXO × Lifting style*).

(a)			Knee Force *Control* [N]		Knee Force *EXO* [N]		Difference (*EXO-Control*)	
Work Task	Parameter	Trunk Orient	Median	(IQR)	Median	(IQR)	[N]	%
Static	HOR_50_	ipsi	57.63	(67.71)	60.11	(122.75)	2.48	4.3%
		front	73.32	(44.90)	88.02	(70.05)	14.69	20.0%
		cont	20.73	(23.37)	−5.47	(48.34)	**−26.19** **^λ^**	**−126.4%**
	VERT_50_	ipsi	896.48	(423.04)	904.65	(433.03)	8.17	0.9%
		front	765.16	(202.38)	839.40	(232.69)	74.24	9.7%
		cont	307.59	(168.52)	349.64	(173.52)	42.05	13.7%
Dynamic	HOR_50_	ipsi	67.12	(96.00)	56.41	(109.21)	−10.70	−15.9%
		front	69.63	(84.01)	59.84	(105.20)	−9.78	−14.1%
		cont	29.84	(41.02)	3.53	(53.79)	**−26.30** **^σ^**	**−88.2%**
	HOR_90_	ipsi	365.43	(326.53)	371.04	(346.22)	5.62	1.5%
		front	287.50	(178.97)	309.12	(224.50)	21.62	7.5%
		cont	95.25	(115.99)	78.93	(110.65)	−16.32	−17.1%
	VERT_50_	ipsi	767.35	(418.68)	806.37	(413.84)	39.03	5.1%
		front	652.35	(313.27)	696.94	(300.28)	44.59	6.8%
		cont	409.13	(243.75)	421.87	(233.23)	12.74	3.1%
	VERT_90_	ipsi	1406.16	(745.10)	1439.82	(723.97)	33.66	2.4%
		front	1009.79	(529.94)	1057.44	(499.05)	47.65	4.7%
		cont	798.45	(306.43)	809.17	(306.52)	10.72	1.3%
**(b)**			**Knee force *Control*** **[N]**		**Knee force *EXO*** **[N]**		**Difference** **(*EXO-Control*)**	
**Work Task**	**Parameter**	**Lifting Style**	**Median**	**(IQR)**	**Median**	**(IQR)**	**[N]**	**%**
Dynamic	HOR_50_	Squat	90.30	(107.91)	61.75	(112.25)	**−28.55** **^σ^**	**−31.6%**
		Stoop	71.15	(102.42)	75.53	(149.61)	4.39	6.2%
	HOR_90_	Squat	301.63	(338.34)	261.76	(358.53)	−39.87	−13.2%
		Stoop	274.26	(286.83)	303.18	(349.62)	28.91	10.5%
	VERT_50_	Squat	613.48	(354.58)	653.30	(376.70)	39.82	6.5%
		Stoop	822.45	(681.98)	840.92	(658.81)	18.47	2.2%
	VERT_90_	Squat	1006.89	(451.76)	1054.35	(481.07)	47.45	4.7%
		Stoop	1343.11	(823.42)	1322.57	(771.82)	−20.53	−1.5%

Significant differences for the post hoc analyses are shown in bold (*p*-values α ≤ 0.00333 for *E* × *TO* and α ≤ 0.00833 for *E* × *LS*). Effect sizes (**^λ^** large effect size (*d* ≥ 0.8); **^σ^** small effect size (*d* ≥ 0.2)) are shown for the significant differences. Detailed statistics are displayed in Appendix B. N = newton; Trunk Orient = *Trunk orientation;* HOR_50_ = 50th percentile of the horizontal force; HOR_90_ = 90th percentile of the horizontal force; VERT_50_ = 50th percentile of the vertical force; VERT_90_ = 90th percentile of the vertical force; ipsi = *ipsilateral*; front = *frontal*; cont = *contralateral*.

**Table 3:** Median knee force values and corresponding interquartile ranges (IQR), absolute and relative differences showing *EXO* compared to *Control* for the dynamic work task (three-fold interactions *EXO × Trunk orientation × Lifting style*).

Parameter	Lifting Style	Trunk Orient	Knee Force *Control* [N]		Knee Force *EXO* [N]		Difference (*EXO-Control*)	
			Median	(IQR)	Median	(IQR)	[N]	%
HOR_50_	Squat	ipsi	126.78	(142.73)	101.62	(146.48)	**−25.15**	**−19.8%**
		front	101.04	(91.74)	74.35	(92.33)	−26.69	−26.4%
		cont	50.36	(65.39)	19.57	(69.46)	**−30.79** **^σ^**	**−61.1%**
	Stoop	ipsi	94.59	(117.18)	107.05	(159.15)	12.46	13.2%
		front	118.36	(90.48)	142.74	(124.94)	**24.39** **^μ^**	**20.6%**
		cont	29.43	(33.49)	4.26	(58.56)	**−25.17** **^μ^**	**−85.5%**
HOR_90_	Squat	ipsi	509.26	(462.72)	481.07	(404.54)	−28.19	−5.5%
		front	284.71	(173.79)	258.84	(238.40)	−25.87	−9.1%
		cont	151.54	(232.13)	109.48	(160.99)	−42.06	−27.8%
	Stoop	ipsi	361.67	(233.48)	406.50	(313.69)	44.83	12.4%
		front	346.07	(133.70)	392.77	(169.43)	46.70	13.5%
		cont	70.81	(67.34)	60.19	(88.21)	−10.61	−15.0%
VERT_50_	Squat	ipsi	833.72	(360.54)	891.83	(325.38)	58.10	7.0%
		front	623.58	(225.56)	667.24	(236.27)	43.66	7.0%
		cont	393.07	(290.35)	387.06	(261.02)	−6.00	−1.5%
	Stoop	ipsi	1125.73	(771.99)	1142.44	(708.87)	16.72	1.5%
		front	1006.42	(431.70)	1022.66	(423.27)	16.24	1.6%
		cont	413.25	(324.03)	439.88	(323.31)	26.63	6.4%
VERT_90_	Squat	ipsi	1319.08	(557.25)	1387.66	(541.02)	68.58	5.2%
		front	933.70	(363.33)	976.06	(373.88)	42.36	4.5%
		cont	864.42	(365.11)	868.97	(335.23)	4.56	0.5%
	Stoop	ipsi	1912.96	(760.15)	1858.51	(796.44)	−54.45	−2.8%
		front	1396.92	(510.29)	1399.91	(467.59)	2.99	0.2%
		cont	856.06	(364.26)	868.64	(359.74)	12.58	1.5%

Significant differences of the post hoc analyses are shown in bold (*p*-value α ≤ 0.00076 for *E × TO × LS*). Effect sizes (**^μ^** medium effect size (*d* ≥ 0.5); **^σ^** small effect size (*d* ≥ 0.2)) are shown for the significant differences. Detailed statistics are displayed in Appendix B. N = newton; *Trunk Orient* = *Trunk orientation;* HOR_50_ = 50th percentile of the horizontal force; HOR_90_ = 90th percentile of the horizontal force; VERT_50_ = 50th percentile of the vertical force; VERT_90_ = 90th percentile of the vertical force; ipsi = *ipsilateral*; front = *frontal*; cont = *contralateral*.

**Table 4:** Median values and corresponding interquartile ranges (IQR) showing the support moment provided by the exoskeleton.

Support Moment [Nm]	Trunk Orient	Static Task		Squat Lifting		Stoop Lifting	
		Median	(IQR)	Median	(IQR)	Median	(IQR)
50th Percentile	ipsi	22.72	(7.26)	19.94	(13.21)	19.05	(16.21)
	front	23.24	(4.81)	20.79	(15.19)	20.93	(16.90)
	cont	22.72	(7.26)	19.94	(13.21)	19.05	(16.21)
90th Percentile	ipsi	NA	NA	29.15	(11.97)	30.25	(10.43)
	front	NA	NA	32.04	(10.61)	32.23	(11.09)
	cont	NA	NA	29.15	(11.97)	30.25	(10.43)

Trunk Orient = *Trunk orientation*; Nm = Newtonmeter; ipsi = *ipsilateral*; front = *frontal*; cont = *contralateral*.

**Figure A1:**
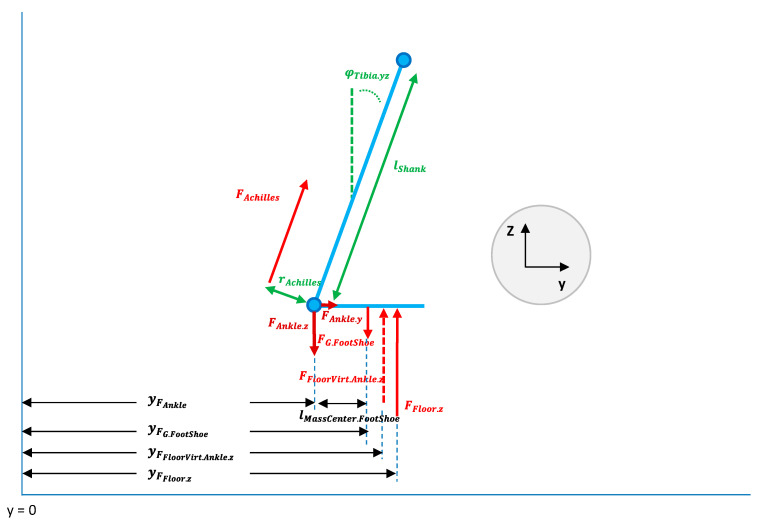
Calculation of ankle joint forces.

**Figure A2:**
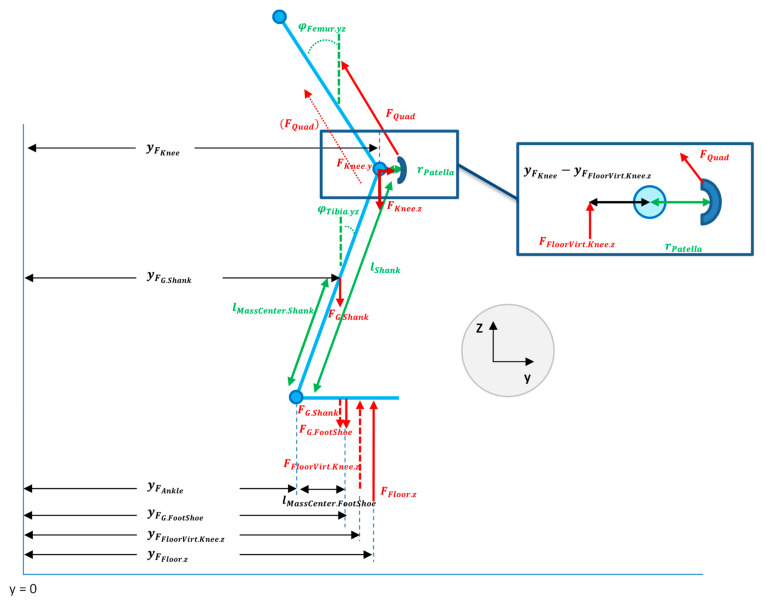
Calculation of knee joint forces.

**Figure A3:**
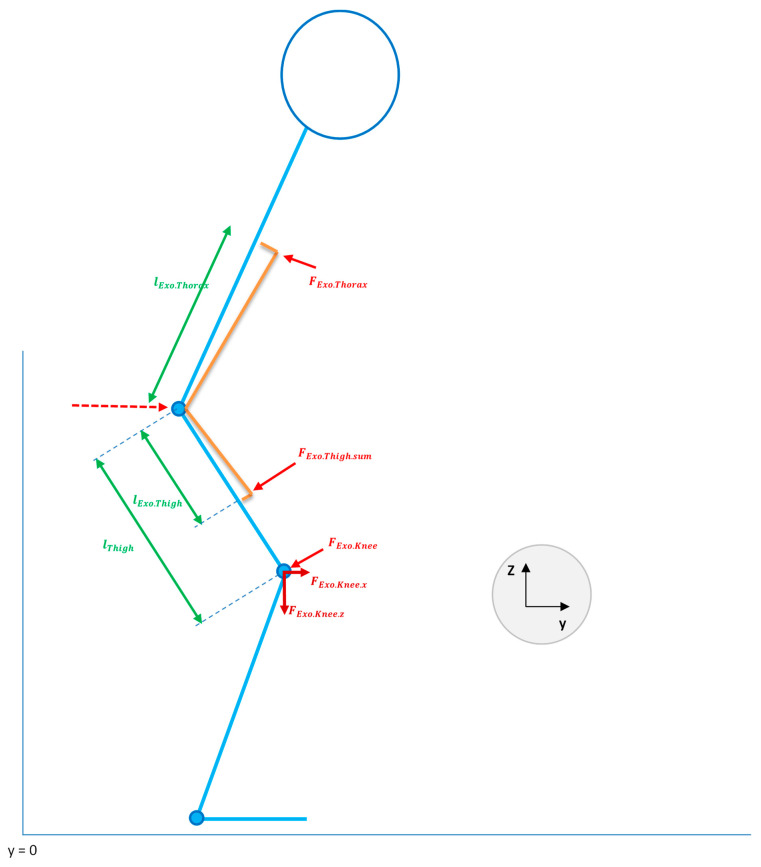
Calculations of knee joint forces including the contribution of the exoskeleton.

## Data Availability

The data are not publicly available due to data use restrictions contained in study participants’ information material.

## Appendix A. Inverse Quasi-Static Model Used for the Knee Force Calculations

**Table A1:** Selection of variables used for the model (forces, coordinates, angles, anthropometrics and segment measures of the subjects).

Ground reaction force (GRF) in three directions [N]: $\mathrm{Horizontal}—\mathrm{mediolateral}\;(x-\mathrm{direction})\;(F_{Floor.x}$) $\mathrm{Horizontal}—\mathrm{anteroposterior}\;(y-\mathrm{direction})\;(F_{Floor.y}$) $\mathrm{vertical}\;(z-\mathrm{direction})\;(F_{Floor.z}$)	Force plate system linked to a signal conditioner and digitizer (FP9090-15-1000; Analog and Digital Amplifier AM6800; resulting resolution 0.5 N and 125 ms in time; Overall maximum error ≤ 6 N *; Bertec Corporation, Columbus, OH, USA)
Ground reaction force vector coordinates (*x*, *y*) [mm] $\left(x_{F_{Floor.z}}\right)$, $\left(y_{F_{Floor.z}}\right)$	$x_{F_{Floor.z}}=-M_{y}/F_{z}$ $y_{F_{Floor.z}}=M_{x}/F_{z}$
$\mathrm{Distribution}\;\mathrm{of}\;\mathrm{the}\;\mathrm{vertical}\;\mathrm{ground}\;\mathrm{reaction}\;\mathrm{force}\;(F_{Floor.z\;}$) onto both feet [N]	The vertical ground reaction force vector on the force plate was recorded continuously, and the *x* and *y* coordinates of both ankle joints were kept constant during the experiments (see explanation above). The total ground reaction force was distributed over both feet using an equation estimating the proportion to the ground reaction force of each foot.$F_{Floor.z.RightLeg}=\frac{F_{Floor.z\left(RightLeg+LeftLeg\right)}*d_{L}}{\left(d_{R}+d_{L}\right)}$ $F_{Floor.z.LeftLeg}=\frac{F_{Floor.z\left(RightLeg+LeftLeg\right)}*d_{R}}{\left(d_{R}+d_{L}\right)}$ $\mathrm{Following},\;\mathrm{both}\;\mathrm{legs}\;(\mathrm{left}\;\mathrm{and}\;\mathrm{right})\;\mathrm{being}\;\mathrm{considered}\;\mathrm{separately}\;\mathrm{in}\;\mathrm{the}\;\mathrm{model}.\;\mathrm{To}\;\mathrm{simplify},\;\mathrm{the}\;\mathrm{terms}\;F_{Floor.z.LeftLeg}$ $\mathrm{and}\;F_{Floor.z.RightLeg}$ $\mathrm{will}\;\mathrm{be}\;\mathrm{omitted}\;\mathrm{and}\;\mathrm{only}\;\mathrm{the}\;\mathrm{formula}\;F_{Floor.z}$ will be used.
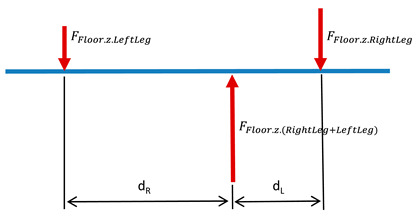
Coordinates (*x*, *y*) of both ankle joint centers of the subjects during the experiments standing on the force plate [mm] $\left(x_{F_{Ankle}}\right)$, $\left(y_{F_{Ankle}}\right)$	The position of both feet was measured and marked prior to the experiments and controlled to always stay in the preset position (Cf. manuscript Section 2.4 Experimental procedure and tasks and Figure 2). The *y*-position of both, the left and right forefoot was assured to be equal. The *x*- and *y*- coordinates of the lateral and medial malleolus were marked on the force plate and the midpoint of the tie line connecting these two points was calculated and used to estimate the *x*- and *y*- coordinates of the ankle joints.
Force of the Laevo^®^ chest pad against the subject’s sternum [µV] $(F_{Exo.Thorax}$)	Measured by a force sensor manually integrated in the chest pad (diameter 38 × thickness 10 mm; Type KM38-1 kN, ME-Messsysteme GmbH, Henningsdorf, Germany), connected to a sampling and storage device (PS12-II; Resolution: 0.1 N; estimated typical error: 0.5 N; maximum error: 1 N) An occurring measurement error depends mainly on undesirable shear forces and undesirable moments acting on the sensor. Estimated typical and maximum measurement errors were determined using known applied forces, shear forces and moments. The shear forces and moments that actually occur during the test were estimated in a qualified manner.
Inclination angles of femur and tibia relative to the perpendicular [°] $(\varphi_{Femur.yz}),\;\left(\varphi_{Tibia.yz}\right)$	Measured by gravimetric inclination sensors connected to a sampling and storage device (PS12-II with 2.5D-gravimetrical sensors; THUMEDI GmbH & Co. KG, resolution 0.1° and 125 ms in time; maximum static error 0.5°; maximum repetition error 0.2°)
Body mass [kg]	Measured with a scale prior to the experiment at the subjects’ first visit in our lab; similar clothing was worn as in the experiment.
Body height [mm]	Measured during an upright stance with the back straight against a wall, feet hip width apart, facing straight ahead.
Partial foot length (distance of the medial sesamoid and malleolus) [mm] **	Measured between the most prominent points over the medial sesamoid and malleolus.
Shank length [mm] $\left(l_{Shank}\right)$ **	Measured on the lateral outside of the shank between the knee joint gap and the malleolus.
Thigh length [mm] $\left(l_{Thigh}\right)$ **	Measured on the lateral outside of the thigh between the knee joint gap and the trochanter major.
Foot mass [kg] $(m_{foot}$)	Foot mass=body mass∗0.000069+0.47 [33]
Foot + shoe mass [kg] $(m_{foot+shoe}$)	Five different sports shoes of different owners were weighed and their relative weight to the foot mass of the owners was calculated. The average relative shoe mass was 0.3229. To estimate the total mass of foot plus shoe, the previously estimated foot mass was multiplied by factor 1.3229.
$\mathrm{Foot}+\mathrm{shoe}\;\mathrm{weight}\;[N]\;\;(F_{G.FootShoe\;}$)	$F_{G.FootShoe\;}=\left(Foot\;mass+shoe\;mass\right)*9.81$
$\mathrm{Shank}\;\mathrm{weight}\;[N]\;(F_{G.Shank})$	$F_{G.Shank}=\left(body\;mass*0.0375+0.38\right)*9.81$ [33]
$\mathrm{Distance}\;\mathrm{between}\;\mathrm{the}\;\mathrm{ankle}\;\mathrm{joint}\;\mathrm{center}\;\mathrm{and}\;\mathrm{the}\;\mathrm{mass}\;\mathrm{center}\;\mathrm{of}\;\mathrm{the}\;\mathrm{shank}\;[\mathrm{mm}]\;(l_{MassCenter.Shank})$	$l_{MassCenter.Shank}=l_{Shank}*0.56$ [32]
Distance between the ankle joint and the center of mass (COM) of the foot (including the shoe) [mm] $\left(l_{MassCenter.FootShoe}\right)$	$l_{MassCenter.FootShoe}={Partial\;foot\;length}_{\left(malleolus-sesamoid\right)}*0.5$ [32]
Radius ankle center to Achilles tendon [mm] $(r_{Achilles}$)	$r_{\mathrm{Achilles}}=body\;height*0.0271$ The factor 0.0271 was estimated by taking measurements of ten male subjects: measuring the distance of the virtual tangent lines of the front- and backside of the ankle joint (on malleolus level). The distance was divided by 2 and relatively related to the individual subjects’ body height. The average of the relative factor of the 10 subjects was calculated and used as factor.
$\mathrm{Radius}\;\mathrm{knee}\;\mathrm{center}\;\mathrm{to}\;\mathrm{Patella}\;[\mathrm{mm}]\;(r_{Patella}$)	$r_{Patella}=body\;height*0.0358$ The factor 0.0358 was estimated by taking measurements of ten male subjects: measuring the distance of a virtual tangent line of the Patella to a virtual tangent line of knee back side. The distance was divided by 2 and relatively related to the individual subjects’ body height. The average of the relative factor of the 10 subjects was calculated and used as factor.
Relevant measures of the Laevo^®^ exoskeleton	Distance smart joint to leg pad: 200 mm Distance smart joint to chest pad: 405 mm (S-size)/435 mm (L-size)

M = moment; F = force/ground reaction force; r = radius; l = length; d = distance; L = left; R = right. * The overall maximum error of GRF was estimated to be ≤ 6 N. Multiple tests were carried out during the measurement periods and always showed an error below 4 N. ** For all measurements of one individual segment (lower limbs), we measured either the left or the right body side, which was randomized to be evaluated and therefore prepared with the measurement equipment, i.e., inclination sensors, in each individual.

### Appendix A.1. Description of the Model

#### Appendix A.1.1. Ankle Joint Forces

Forces and moments acting on the ankle joint are calculated, including the vertical and horizontal forces acting on the ankle joint ($F_{Ankle.z\;}$ and $F_{Ankle.y\;}$, respectively) and on the Achilles tendon ($F_{Achilles})$. Therefore, the y-coordinates of the vertical ground reaction force $(y_{F_{Floor.z}}$), the y-coordinates of both ankle joints $(y_{F_{Ankle}}$), the perpendicular distance between the pivot point of the ankle joint and the force vector of the Achilles tendon ($r_{Achilles}$) were used. The dead weight of the feet, including shoes ($F_{G.FootShoe\;}$), was considered to not contributing to the load on the ankle joint. To simplify the model, it is assumed that $F_{Achilles}$ acts parallel to the shank.

**Table A2:** Symbols with description and Equations as used for the model displayed in Figure A1.

Symbol	Description
$\varphi_{Tibia.yz}$	Angle between tibia and the perpendicular (in *y*/*z*-direction).
$l_{Shank}$	Shank length.
$F_{Achilles}$	Forces acting on the Achilles tendon.
$r_{Achilles}$	Radius of ankle joint center to Achilles tendon.
$F_{Ankle.z}$	Force acting on the ankle joint in *z*-direction.
$F_{Ankle.y}$	Force acting on the ankle joint in *y*-direction.
$F_{G.FootShoe}$	Segment weight of foot + shoe.
$F_{FloorVirt.Ankle.z}$	Virtual ground reaction force in *z*-direction, excluding foot + shoe mass.
$F_{Floor.z\;}$	Ground reaction force in *z*-direction.
$y_{F_{Ankle}}$	Y-position of the ankle joint.
$y_{F_{G.FootShoe}}$	Y-position of the force vector of the foot + shoe center of mass.
$y_{F_{FloorVirt.Ankle.z}}$	Y-position of the virtual ground reaction force vector, excluding foot + shoe mass.
$y_{F_{Floor.z}}$	Y-position of the total ground reaction force vector.
$l_{MassCenter.FootShoe}$	Distance between the ankle joint and the mass center of foot + shoe.
**Symbol**	**Equation**
$F_{FloorVirt.Ankle.z}$	$=F_{Floor.z}-F_{G.FootShoe}$
$y_{F_{FloorVirt.Ankle.z}}$	$=\frac{F_{Floor.z}\cdot y_{F_{Floor.z}}-F_{G.FootShoe}\cdot y_{F_{G.FootShoe}}}{F_{FloorVirt.Ankle.z}}$
$y_{F_{G.FootShoe}}$	$=y_{F_{Ankle}}+l_{MassCenter.FootShoe}$
$F_{Achilles}$	$=F_{FloorVirt.Ankle.z}\cdot \frac{y_{F_{FloorVirt.Ankle.z}}-y_{F_{Ankle}}}{r_{Achilles}}$
$F_{Ankle.z}$	$=F_{FloorVirt.Ankle.z}+cos\left(\varphi_{Tibia.yz}\right)\cdot F_{Achilles}\;$ $=F_{Floor.z}-F_{G.FootShoe}+cos\left(\varphi_{Tibia.yz}\right)\cdot F_{Achilles}$ $=F_{Floor.z}-F_{G.FootShoe}+cos\left(\varphi_{Tibia.yz}\right)\cdot \left(F_{Floor.z}-F_{G.FootShoe}\right)\cdot \frac{y_{F_{FloorVirt.Ankle.z}}-y_{F_{Ankle}}}{r_{Achilles}}$
$F_{Ankle.y}$	$=sin\left(\varphi_{Tibia.yz}\right)\cdot F_{Achilles}$ $=sin\left(\varphi_{Tibia.yz}\right)\cdot \left(F_{Floor.z}-F_{G.FootShoe}\right)\cdot \frac{y_{F_{FloorVirt.Ankle.z}}-y_{F_{Ankle}}}{r_{Achilles}}$
$F_{Ankle.y}$	$=sin\left(\varphi_{Tibia.yz}\right)\cdot F_{Achilles}$ $=sin\left(\varphi_{Tibia.yz}\right)\cdot \left(F_{Floor.z}-F_{G.FootShoe}\right)\cdot \frac{y_{F_{FloorVirt.Ankle.z}}-y_{F_{Ankle}}}{r_{Achilles}}$
$F_{Ankle.yz}$	$=\sqrt{F_{Ankle.z}^{2}+F_{Ankle.y}^{2}}$
$\varphi \_F_{Ankle.yz}$	$=arctan\left(\frac{F_{Ankle.y}}{F_{Ankle.z}}\right)$

#### Appendix A.1.2. Knee Joint Forces

The ratio of the weight of the shanks contributing to its segment weight $(F_{G.Shank}$) and the coordinates of these force vectors $(y_{F_{G.Shank}}$) is calculated using the y-coordinates of the ankle joints ($y_{F_{Ankle}}$), the inclination angles of the tibia ($\varphi_{Tibia.yz})$, the shanks’ weight and the distance between ankle and shanks’ center of gravity ($l_{MassCenter.Shank})$. These force components should be considered because they act on the body below the knees and therefore do not contribute to the force acting on the knee joints.

A resulting “virtual ground reaction force vector”, excluding shank, foot and shoe ($F_{FloorVirt.Knee.z\;})$, is of importance for the calculation of the forces and moments acting on the knee joints.

In a next step, the force acting on the quadriceps tendon ($F_{Quad}$) is calculated. For this purpose, the coordinates of the knee joints $(y_{F_{Knee}}$), which were estimated using the shank’s inclination angle in the sagittal plane ($\varphi_{Tibia.yz}$) and the shank’s length $(l_{Shank}$), the $F_{FloorVirt.Knee.z\;}$, and the distance between the patella and the rotation axis of the knee joint ($r_{Patella}$) were used for the model. To simplify the model, we assume ${\vec{F}}_{Quad}$ to act parallel to the shank. Further, the knee joint moments and the vertical ($F_{Knee.z\;}$), horizontal ($F_{Knee.y}$) and resulting total forces acting on the knee joints are calculated using the $F_{Quad}$, $F_{FlorVirt.Knee,z}$, and the inclination angle of the femur ($\varphi_{Femur.yz}$).

**Table A3:** Symbols with description and Equations as used for the model displayed in Figure A2.

Symbol	Description
$\varphi_{Femur.yz}$	= Angle between femur and the perpendicular (*y*/*z*-direction)
$F_{Quad}$	= Force acting at the Quadriceps tendon
$r_{Patella}$	= Radius of knee joint center to patella
$F_{Knee.z\;}$	= Force at the knee joint in *z*-direction (without exoskeleton)
$F_{Knee.y\;}$	= Force at the knee joint in *y*-direction (without exoskeleton)
$\varphi_{Tibia.yz}$	= Angle between tibia and the perpendicular (in *y*/*z*-direction)
$l_{Shank}$	= Shank length
$l_{MassCenter.Shank}$	= Distance between the ankle joint and the mass center of the shank
$F_{G.Shank}$	= Segment weight of the shank
$F_{FloorVirt.Knee.z\;}$	= Virtual ground reaction force in *z*-direction, excluding foot + shoe + shank mass
$F_{G.FootShoe}$	= Segment weight of foot + shoe
$F_{Floor.z\;}$	= Ground reaction force in *z*-direction
$y_{F_{Knee}}$	= Y-position of the knee joint
$y_{F_{G.Shank}}$	= Y-position of the force vector of the shank center of mass
$y_{F_{Ankle}}$	= Y-position of the ankle joint
$y_{F_{G.FootShoe}}$	= Y-position of the force vector of the foot + shoe center of mass
$y_{F_{FloorVirt.Knee.z}}$	= Y-position of the virtual ground reaction force vector, excluding foot + shoe and shank mass
$y_{F_{Floor.z}}$	= Y-position of the total ground reaction force vector
$l_{MassCenter.FootShoe}$	= Distance between the ankle joint and the mass center of foot + shoe
**Symbol**	**Equation**
$F_{FloorVirt.Knee.z}\;\;\;\;\;\;\;\;\;\;\;$	$=F_{FloorVirt.Ankle.z}-F_{G.Shank}=F_{Floor.z}-F_{G.FootShoe}-F_{G.Shank}$
$y_{F_{FloorVirt.Knee.z}}$	$=\frac{F_{Floor.z}\cdot y_{F_{Floor.z}}-F_{G.Shank}\cdot y_{F_{G.Shank}}-F_{G.FootShoe}\cdot y_{F_{G.FootShoe}}}{F_{FloorVirt.Knee.z}}$
$y_{F_{Knee}}$	$=y_{F_{Ankle}}+sin\left(\varphi_{Tibia.yz}\right)\cdot l_{Shank}$
$y_{F_{G.Shank}}$	$=y_{F_{Ankle}}+sin\left(\varphi_{Tibia.yz}\right)\cdot l_{MassCenter.Shank}$
$y_{F_{G.FootShoe}}$	$=y_{F_{Ankle}}+l_{MassCenter.FootShoe}$
$F_{Quad}$	$=F_{FloorVirt.Knee.z}\cdot \frac{y_{F_{Knee}}-y_{F_{FloorVirt.Knee.z}}}{r_{Patella}}$ $=\left(F_{Floor.z}-F_{G.FootShoe}-F_{G.Shank}\right)\cdot \ldots$ $\ldots \frac{\left|y_{F_{Ankle}}+sin\left(\varphi_{Tibia.yz}\right)\cdot l_{Shank}-\frac{F_{Floor.z}\cdot y_{F_{Floor.z}}-F_{G.Shank}\cdot \left(y_{F_{Ankle}}+sin\left(\varphi_{Tibia.yz}\right)\cdot l_{MassCenter.Shank}\right)-F_{G.FootShoe}\cdot \left(y_{F_{Ankle}}+l_{MassCenter.FootShoe}\right)}{F_{Floor.z}-F_{G.FootShoe}-F_{G.Shank}}\right|}{r_{Patella}}$
$F_{Knee.z}\;$	$=F_{FloorVirt.Knee.z}+cos\left(\varphi_{Femur.yz}\right)\cdot F_{Quad}$ $=F_{Floor.z}-F_{G.FootShoe}-F_{G.Shank}+cos\left(\varphi_{Femur.yz}\right)\cdot F_{Quad}$
$F_{Knee.y}\;\;\;\;\;\;\;$	$=sin\left(\varphi_{Femur.yz}\right)\cdot F_{Quad}$
$F_{Knee.yz}\;$	$=\sqrt{F_{Knee.z}^{2}+F_{Knee.y}^{2}}$
$\varphi \_F_{Knee.yz}$	$=arctan\left(\frac{F_{Knee.y}}{F_{Knee.z}}\right)$

Force acting on the knee joint is induced by the thigh muscles pulling on the anterior and posterior sides (simplified in this model); therefore, we calculated the absolute value of the “$y_{F_{Knee}}-y_{F_{FloorVirt.Knee.z}}”$ within the $“F_{Quad}”$-calculation. Essential is that the most of the force is acting on the front side of the knee joint, being transmitted from the quadriceps muscle to the quadriceps tendon. Therefore, the designation “$F_{Quad}$” was chosen.

#### Appendix A.1.3. Contribution of the Laevo® Exoskeleton

The Laevo^®^ (Laevo^®^, Delft, The Netherlands) exoskeleton generates a moment about its rotation axis (“smart joint”) passing through the hip joints, assuming the rotating axis of the exoskeleton equals the axis which is passing through the rotation points of the hip joints. Therefore, the exoskeleton is loading the exoskeletons’ contact points, e.g., the chest at sternum level, the upper legs and the pelvis. The amount of force is determined by (1) the inclination angle of the exoskeletons “smart joint”, i.e., the angle between the trunk and the thighs, and by (2) the movement direction (e.g., upwards or downwards). The exoskeleton’s support moment ($M_{Exo}$) and the force acting on the thighs ($F_{Exo.Femur.sum}$) were recorded using the measurements of the force sensor that was integrated in the exoskeletons’ chest pad ($F_{Exo.Thorax}$) and the geometric dimensions of the exoskeleton’s structures ($l_{Exo.Thorax}$ and $l_{Exo.Thigh}$).

The forces related to the exoskeleton additionally act on the knee joints, being reduced by the thigh that acts as the lever arm. These exoskeleton-related forces were considered in the model as additively overlaying horizontal and vertical components of the forces acting on the knee joints.

**Table A4:** Symbols with description and Equations as used for the model displayed in Figure A3.

Symbol	Description
$l_{Exo.Thorax}\;$	= Distance between the smart joints and the chest pad
$l_{Thigh}\;$	= Thigh length
$l_{Exo.Thigh}$	= Distance between the smart joints and the leg pads
$\varphi_{Femur.yz}$	= Angle between femur and the perpendicular (y/*z*-direction)
$F_{Exo.Thorax\;}$	= Force acting on the chest pad
$F_{Exo.Thigh.sum\;}$	= Force acting on the leg pads
$F_{Exo.Knee}$	= Force acting on the knee induced by the exoskeleton
$F_{Exo.Knee.z\;}$	=Force acting on the knee induced by the exoskeleton in z-direction
$F_{Exo.Knee.y\;}$	= Force acting on the knee induced by the exoskeleton in *y*-direction
$F_{KneeWithExo.z\;}$	= Total force at the knee joint in *z*-direction (exoskeleton included)
$F_{KneeWIthExo.y\;}$	= Total force at the knee joint in *y*-direction (exoskeleton included)
**Symbol**	**Equation**
$M_{Exo}\;\;\;\;\;\;\;$	$=F_{Exo.Thorax}\cdot l_{Exo.Thorax}$
$F_{Exo.Thigh.sum}$	$=\frac{F_{Exo.Thorax}\cdot l_{Exo.Thorax}}{l_{Exo.Thigh}}=\frac{M_{Exo}}{l_{Exo.Thigh}}$
$F_{Exo.Thigh\;}$	$=\;\frac{F_{Exo.Thigh.sum}}{2}$
$F_{Exo.Knee}$	$=\frac{F_{Exo.Thigh.\frac{l}{r}}\cdot l_{Exo.Thigh}}{l_{Thigh}}=\frac{F_{Exo.Thorax}\cdot l_{Exo.Thorax}\cdot l_{Exo.Thigh}}{l_{Exo.Thigh}\cdot l_{Thigh}}$ $=\frac{F_{Exo.Thorax}\cdot l_{Exo.Thorax}}{l_{Thigh}}=\frac{M_{Exo}}{l_{Thigh}}$
$F_{Exo.Knee.z}$	$=\frac{F_{Exo.Thorax}\cdot l_{Exo.Thorax}}{l_{Thigh}}\cdot sin\left(\varphi_{Femur.yz}\right)$ $=F_{Exo.Knee}\cdot sin\left(\varphi_{Femur.yz}\right)$
$F_{Exo.Knee.y}$	$=-\frac{F_{Exo.Thorax}\cdot l_{Exo.Thorax}}{l_{Thigh}}\cdot cos\left(\varphi_{Femur.yz}\right)$ $=-F_{Exo.Knee}\cdot cos\left(\varphi_{Femur.yz}\right)$
$F_{KneeWithExo.z}\;\;\;\;\;\;$	$=F_{Floor.z}-F_{G.FootShoe}-F_{G.Shank}+cos\left(\varphi_{Femur.yz}\right)\cdot F_{Thigh}\ldots$ $+\frac{F_{Exo.Thorax}\cdot l_{Exo.Thorax}}{l_{Thigh}}\cdot sin\left(\varphi_{Femur.yz}\right)$ $=F_{Knee.z}+F_{Exo.Knee.z}$
$F_{KneeWithExo.y}\;\;\;\;\;\;$	$=sin\left(\varphi_{Femur.yz}\right)\cdot F_{Thigh}$$-\frac{F_{Exo.Thorax}\cdot l_{Exo.Thorax}}{l_{Thigh}}\cdot cos\left(\varphi_{Femur.yz}\right)$ $=F_{Knee.y}+F_{Exo.Knee.y}$
$F_{KneeWithExo.yz}\;\;\;\;\;$	$=\sqrt{F_{KneeWithExo.z}^{2}+F_{KneeWithExo.y}^{2}}$
$\varphi \_F_{KneeWithExo.yz}$	$=arctan\left(\frac{F_{KneeWithExo.y}}{F_{KneeWithExo.z}}\right)$

#### Appendix A.1.4. Limitations

Neglecting the dynamic movements

- Cf. Section 4.1 Limitations (manuscript)

Neglecting the horizontal ground reaction forces in this model:

- In this model, solely the vertical component (*z*-direction) of the ground reaction force is integrated; both horizontal acting components (x- and y-direction) of the ground reaction force are presumed to be extremely low in the here presented work tasks due to the experimental design. (No horizontal movements of the lower limbs and no fast movements have been included. Horizontal forces may only occur for short moments and to a small extent, due to the mass inertia during movement.)

Distribution of the exoskeleton’s force acting onto the thighs

- The calculated sum of the force generated by the exoskeleton and acting on the subject’s thighs was distributed onto both legs to be loaded by 50% each. This simplification of the model was applied instead of considering the individual angles between each upper leg and the trunk, because only one leg was prepared with the measurement equipment. Assuming that the subjects bent their trunk relative to their upper limbs similar between both body sides, the resulting division of the forces were exact. The expected error according to this simplification may be minimal since the main contribution to the knee joint loading is caused by the subjects’ body weight and not by the exoskeleton.

Calculation of $r_{Achilles}$ and $r_{Patella}$

- We did not find any standard terms for calculating or estimating the $r_{Achilles}$ and $r_{Patella}$ in the available literature; therefore, we used own measurements for calculating an average ratio of the radius $r_{Achilles}$ and $r_{Patella}$ in relation to body size. Errors might result from this simplification in our model since the real individual distances were not considered. However, we used a within-subject-design, comparing the different conditions within each subject. Therefore, the relative changes between conditions can be used for comparison.

Joints and joint forces

- The ankle and knee joints were treated like a simple hinge joints; other aspects of the joint functions and movement variances were neglected.
- Torsional forces were neglected. However, the Laevo^®^ is not designed to support torsional forces and is unable to absorb those. Therefore, the exoskeleton should not have any impact on torsional forces at the knee joints.
- The pivot point of a joint is not necessarily central which is assumed in this model. Further, when a body is moving the pivot point of a joint is shifting. The virtual pivot point of the joints is neglected in the model.
- The model includes forces which are produced by muscles responsible for the main movement in the work task (agonists). Forces which are induced by the antagonistic muscles (e.g., the biceps femoris) are not considered. Kellis and Baltzopoulos [58] showed an influence of including the antagonistic (hamstrings) muscle force into a two-dimensional tibiofemoral joint force model which increased the posteriorly directed shear and compression forces. In our force estimating model the antagonistic muscle force has only been partly included (calf muscle forces but not hamstrings), which could have biased the calculated knee forces.

Gravimetric position sensors

- Gravimetric positions sensors provide a very high precision in static postures and slower movements (standard error ≤ 0.5°). In fast movements including high accelerations (which were not included in this experiments) these sensors are less precise; other techniques (i.e., motion capture systems) should then be applied.

## Appendix B

**Table A5:** *F*-values and *p*-values of the repeated measures ANOVAs with corresponding effect sizes (partial eta squared ($\eta_{p}^{2}$)). Main effects of the *Exoskeleton* condition (*E*) and the interaction effects for *E* with *Trunk orientation* (*E × TO*), *E* with *Lifting style* (*E × LS*) and *E* with *TO* and *LS* (*E × TO × LS*) for static and dynamic work tasks.

Task	Effect	HOR_50_			HOR_90_			VERT_50_			VERT_90_		
		*F*	*p*	$\eta_{p}^{2}$	*F*	*p*	$\eta_{p}^{2}$	*F*	*p*	$\eta_{p}^{2}$	*F*	*p*	$\eta_{p}^{2}$
Static	*E*	0.16	0.696	0.006	-	-	-	7.41	**0.011**	**0.209**	-	-	-
	*E × TO*	27.60	**<0.001 ***	**0.496** **^λ^**				1.19	0.313	**0.041**			
Dynamic	*E*	7.24	**0.012 ***	**0.205** **^μ^**	0.02	0.888	0.001	16.85	**<0.001 ***	**0.376** **^λ^**	1.64	0.211	**0.054** **^σ^**
	*E × TO*	23.34	**<0.001 ***	**0.455** **^λ^**	11.90	**<0.001 ***	**0.292** **^λ^**	1.02	0.368	**0.035** **^σ^**	0.16	0.852	0.005
	*E × LS*	24.96	**<0.001 ***	**0.471** **^λ^**	8.84	**0.006 ***	**0.236** **^μ^**	1.52	0.227	**0.052** **^σ^**	4.06	0.053	**0.121** **^σ^**
	*E × TO × LS*	7.04	**0.002 ***	**0.201** **^μ^**	1.52	0.227	**0.049** **^σ^**	2.96	0.060	**0.096** **^σ^**	2.60	0.083	**0.086** **^σ^**

* Significant *p*-values (α ≤ 0.05); **^λ^** large effect size ($\eta_{p}^{2}$ ≥ 0.26); **^μ^** medium effect size ($\eta_{p}^{2}$ ≥ 0.13); **^σ^** small effect size ($\eta_{p}^{2}$ ≥ 0.02); HOR_50_ = 50th percentile of the horizontal force; HOR_90_ = 90th percentile of the horizontal force; VERT_50_ = 50th percentile of the vertical force; VERT_90_ = 90th percentile of the vertical force.

**Table A6:** Pairwise comparisons (*p*-values and Cohens’d (*d*)) for the relevant interactions between *E × TO,* *E × LS,* and *E × TO × LS* for variables with significant interaction effects for static and dynamic work tasks.

Task	Effect	Trunk Orient	Lifting Style	HOR_50_		HOR_90_	
				*p*	*d*	*p*	*d*
Stat	*E × TO*	ipsi		0.372	0.083	n.a.	n.a.
		front		0.091	**0.269** **^σ^**	n.a.	n.a.
		cont		**<0.001 ***	**−0.912** **^λ^**	n.a.	n.a.
Dyn	*E × TO*	ipsi		0.311	−0.045	0.981	−0.024
		front		0.834	0.018	0.028	**0.230** **^σ^**
		cont		**<0.001 ***	**−0.493** **^σ^**	0.013	**−0.244** **^σ^**
	*E × LS*		Squat	**<0.001 ***	**−0.261** **^σ^**	0.033	−0.139
			Stoop	0.276	0.072	0.050	0.145
	*E × TO × LS*	ipsi	Squat	**<0.001 ***	**−0.195**	−	−
			Stoop	0.024	0.166	−	−
		front	Squat	0.001	**−0.335** **^σ^**	−	−
			Stoop	**<0.001 ***	**0.597** **^μ^**	−	−
		cont	Squat	**<0.001 ***	**−0.487** **^σ^**	−	−
			Stoop	**<0.001 ***	**−0.717** **^μ^**	−	−

* Significant *p*-values (α ≤ 0.00333) for *E × TO*; (α ≤ 0.00833) for *E × LS*; (α ≤ 0.00076) for *E × TO × LS*; ^λ^ large effect size (*d* ≥ 0.8); **^μ^** medium effect size (*d* ≥ 0.5); **^σ^** small effect size (*d* ≥ 0.2); Trunk Orient = *Trunk orientation*; HOR_50_ = 50th percentile of the horizontal force; HOR_90_ = 90th percentile of the horizontal force; VERT_50_ = 50th percentile of the vertical force; VERT_90_ = 90th percentile of the vertical force.

## References

[1] DeKok J., Vroonhof P., Snijders J., Roullis G., Clarke M., Peereboom K., vanDorst P., Isusi I. (2019). Work-Related Musculoskeletal Disorders: Prevalence, Costs and Demographics in the EU.

[2] Dick R.B., Lowe B.D., Lu M.L., Krieg E.F. (2020). Trends in work-related musculoskeletal disorders from the 2002—2014 General Social Survey, Quality of Work Life supplement. J. Occup. Environ. Med..

[3] Coenen P., Kingma I., Boot C.R., Twisk J.W., Bongers P.M., van Dieen J.H. (2013). Cumulative low back load at work as a risk factor of low back pain: A prospective cohort study. J. Occup. Rehabil..

[4] Steinhilber B., Luger T., Schwenkreis P., Middeldorf S., Bork H., Mann B., von Glinski A., Schildhauer T.A., Weiler S., Schmauder M. (2020). The use of exoskeletons in the occupational context for primary, secondary, and tertiary prevention of work-related musculoskeletal complaints. IISE Trans. Occup. Ergon. Hum. Factors.

[5] Toxiri S., Näf M.B., Lazzaroni M., Fernández J., Sposito M., Poliero T., Monica L., Anastasi S., Caldwell D.G., Ortiz J. (2019). Back-support exoskeletons for occupational use: An overview of technological advances and trends. IISE Trans. Occup. Ergon. Hum. Factors.

[6] Bär M., Steinhilber B., Rieger M.A., Luger T. (2021). The influence of using exoskeletons during occupational tasks on acute physical stress and strain compared to no exoskeleton—A systematic review and meta-analysis. Appl. Ergon..

[7] Koopman A.S., Kingma I., de Looze M.P., van Dieën J.H. (2020). Effects of a passive back exoskeleton on the mechanical loading of the low-back during symmetric lifting. J. Biomech..

[8] Baltrusch S.J., van Dieen J.H., Koopman A.S., Naf M.B., Rodriguez-Guerrero C., Babic J., Houdijk H. (2020). SPEXOR passive spinal exoskeleton decreases metabolic cost during symmetric repetitive lifting. Eur. J. Appl. Physiol..

[9] Alemi M.M., Madinei S., Kim S., Srinivasan D., Nussbaum M.A. (2020). Effects of two passive back-support exoskeletons on muscle activity, energy expenditure, and subjective assessments during repetitive lifting. Hum. Factors.

[10] Madinei S., Alemi M.M., Kim S., Srinivasan D., Nussbaum M.A. (2020). Biomechanical assessment of two back-support exoskeletons in symmetric and asymmetric repetitive lifting with moderate postural demands. Appl. Ergon..

[11] Luger T., Bär M., Seibt R., Rimmele P., Rieger M.A., Steinhilber B. (2021). A passive back exoskeleton supporting symmetric and asymmetric lifting in stoop and squat posture reduces trunk and hip extensor muscle activity and adjusts body posture—A laboratory study. Appl. Ergon..

[12] Bosch T., van Eck J., Knitel K., de Looze M. (2016). The effects of a passive exoskeleton on muscle activity, discomfort and endurance time in forward bending work. Appl. Ergon..

[13] Koopman A.S., Kingma I., Faber G.S., de Looze M.P., van Dieën J.H. (2019). Effects of a passive exoskeleton on the mechanical loading of the low back in static holding tasks. J. Biomech..

[14] Graham R.B., Agnew M.J., Stevenson J.M. (2009). Effectiveness of an on-body lifting aid at reducing low back physical demands during an automotive assembly task: Assessment of EMG response and user acceptability. Appl. Ergon..

[15] Madinei S., Alemi M.M., Kim S., Srinivasan D., Nussbaum M.A. (2020). Biomechanical evaluation of passive back-support exoskeletons in a precision manual assembly task: “Expected” effects on trunk muscle activity, perceived exertion, and task performance. Hum. Factors.

[16] Bär M., Luger T., Seibt R., Rieger M.A., Steinhilber B. (2022). Using a Passive Back Exoskeleton during a Simulated Sorting Task: Influence on Muscle Activity, Posture, and Heart Rate. Hum. Factors.

[17] Theurel J., Desbrosses K. (2019). Occupational exoskeletons: Overview of their benefits and limitations in preventing work-related musculoskeletal disorders. IISE Trans. Occup. Ergon. Hum. Factors.

[18] Kim S., Madinei S., Alemi M.M., Srinivasan D., Nussbaum M.A. (2020). Assessing the potential for “undesired” effects of passive back-support exoskeleton use during a simulated manual assembly task: Muscle activity, posture, balance, discomfort, and usability. Appl. Ergon..

[19] Luger T., Bar M., Seibt R., Rieger M.A., Steinhilber B. (2021). Using a Back Exoskeleton during Industrial and Functional Tasks-Effects on Muscle Activity, Posture, Performance, Usability, and Wearer Discomfort in a Laboratory Trial. Hum. Factors.

[20] Frost D.M., Abdoli E.M., Stevenson J.M. (2009). PLAD (personal lift assistive device) stiffness affects the lumbar flexion/extension moment and the posterior chain EMG during symmetrical lifting tasks. J. Electromyogr. Kinesiol..

[21] Alemi M.M., Geissinger J., Simon A.A., Chang S.E., Asbeck A.T. (2019). A passive exoskeleton reduces peak and mean EMG during symmetric and asymmetric lifting. J. Electromyogr. Kinesiol..

[22] Ulrey B.L., Fathallah F.A. (2013). Effect of a personal weight transfer device on muscle activities and joint flexions in the stooped posture. J. Electromyogr. Kinesiol..

[23] von Glinski A., Yilmaz E., Mrotzek S., Marek E., Jettkant B., Brinkemper A., Fisahn C., Schildhauer T.A., Geßmann J. (2019). Effectiveness of an on-body lifting aid (HAL^®^ for care support) to reduce lower back muscle activity during repetitive lifting tasks. J. Clin. Neurosci..

[24] Steinhilber B.B.M., Caputo G., Seibt R., Rieger M.A., Luger T. (2020). Einfluss eines passiven Exoskeletts zur Rückenunterstützung auf die erlebte körperliche Arbeitsbeanspruchung und Beschwerdewahrnehmung bei simulierten Tätigkeiten mit statischer Oberkörpervorneigung und dynamischen Hebebewegungen. Arb. Soz. Umweltmed. Z. Med. Prävent..

[25] Weston E.B., Alizadeh M., Knapik G.G., Wang X., Marras W.S. (2018). Biomechanical evaluation of exoskeleton use on loading of the lumbar spine. Appl. Ergon..

[26] Govaerts R., Tassignon B., Ghillebert J., Serrien B., De Bock S., Ampe T., El Makrini I., Vanderborght B., Meeusen R., De Pauw K. (2021). Prevalence and incidence of work-related musculoskeletal disorders in secondary industries of 21st century Europe: A systematic review and meta-analysis. BMC Musculoskelet. Disord..

[27] Da Costa B.R., Vieira E.R. (2010). Risk factors for work-related musculoskeletal disorders: A systematic review of recent longitudinal studies. Am. J. Ind. Med..

[28] University Hospital Tübingen Work Physiological-Biomechanical Analysis of a Passive Exoskeleton to Support Occupational Lifting and Flexing Processes (ADVANCE). https://clinicaltrials.gov/ct2/show/NCT03725982.

[29] Bate S.T., Jones B. (2008). A review of uniform cross-over designs. J. Stat. Plan. Inference.

[30] Harari Y., Bechar A., Asci S., Riemer R. (2021). Investigation of 3D dynamic and quasistatic models for spinal moments during combined manual material handling tasks. Appl. Ergon..

[31] Krishnan R.H., Devanandh V., Brahma A.K., Pugazhenthi S. (2016). Estimation of Mass Moment of Inertia of Human Body, When Bending Forward, for the Design of a Self-Transfer Robotic Facility. J. Eng. Sci. Technol..

[32] Winter D.A. (2009). Biomechanics and Motor Control of Human Movement.

[33] NASA National Aeronautics and Space Administration (1978). Anthropometric Source Book.

[34] Kim H.Y. (2013). Statistical notes for clinical researchers: Assessing normal distribution (2) using skewness and kurtosis. Restor. Dent. Endod..

[35] Bakeman R. (2005). Recommended effect size statistics for repeated measures designs. Behav. Res. Methods.

[36] Cohen J. (1988). Statistical Power Analysis for the Behavioral Sciences.

[37] Kaufman K.R., An K.N., Litchy W.J., Morrey B.F., Chao E.Y.S. (1991). Dynamic Joint Forces during Knee Isokinetic Exercise. Am. J. Sport Med..

[38] Nisell R., Ericson M.O., Nemeth G., Ekholm J. (1989). Tibiofemoral Joint Forces during Isokinetic Knee Extension. Am. J. Sport Med..

[39] Miranda H., Viikari-Juntura E., Martikainen R., Riihimaki H. (2002). A prospective study on knee pain and its risk factors. Osteoarthr. Cartil..

[40] Verbeek J., Mischke C., Robinson R., Ijaz S., Kuijer P., Kievit A., Ojajarvi A., Neuvonen K. (2017). Occupational Exposure to Knee Loading and the Risk of Osteoarthritis of the Knee: A Systematic Review and a Dose-Response Meta-Analysis. Saf. Health Work.

[41] D’Lima D.D., Fregly B.J., Patil S., Steklov N., Colwell C.W. (2012). Knee joint forces: Prediction, measurement, and significance. Proc. Inst. Mech. Eng. Part H J. Eng. Med..

[42] D’Lima D.D., Patil S., Steklov N., Chien S., Colwell C.W. (2007). In vivo knee moments and shear after total knee arthroplasty. J. Biomech..

[43] Kutzner I., Heinlein B., Graichen F., Bender A., Rohlmann A., Halder A., Beier A., Bergmann G. (2010). Loading of the knee joint during activities of daily living measured in vivo in five subjects. J. Biomech..

[44] Kutzner I., Stephan D., Dymke J., Bender A., Graichen F., Bergmann G. (2013). The influence of footwear on knee joint loading during walking—In vivo load measurements with instrumented knee implants. J. Biomech..

[45] Taylor S.J., Walker P.S., Perry J.S., Cannon S.R., Woledge R. (1998). The forces in the distal femur and the knee during walking and other activities measured by telemetry. J. Arthroplast..

[46] Taylor W.R., Heller M.O., Bergmann G., Duda G.N. (2004). Tibio-femoral loading during human gait and stair climbing. J. Orthop. Res..

[47] Nagura T., Dyrby C.O., Alexander E.J., Andriacchi T.P. (2002). Mechanical loads at the knee joint during deep flexion. J. Orthop. Res..

[48] Sandmark H., Hogstedt C., Vingard E. (2000). Primary osteoarthrosis of the knee in men and women as a result of lifelong physical load from work. Scand. J. Work Environ. Health.

[49] Coggon D., Croft P., Kellingray S., Barrett D., McLaren M., Cooper C. (2000). Occupational physical activities and osteoarthritis of the knee. Arthritis Rheum..

[50] Klussmann A., Gebhardt H., Nubling M., Liebers F., Perea E.Q., Cordier W., von Engelhardt L.V., Schubert M., David A., Bouillon B. (2010). Individual and occupational risk factors for knee osteoarthritis: Results of a case-control study in Germany. Arthritis Res. Ther..

[51] Christa Walzer M.T. (2016). Berufskrankheiten Durch Mechanische Einwirkung—Raten Bestätigter BK-Fälle in Einzelberufen.

[52] Capin J.J., Khandha A., Zarzycki R., Arundale A.J.H., Ziegler M.L., Manal K., Buchanan T.S., Snyder-Mackler L. (2018). Gait mechanics and tibiofemoral loading in men of the ACL-SPORTS randomized control trial. J. Orthop. Res..

[53] Costigan P.A., Deluzio K.J., Wyss U.P. (2002). Knee and hip kinetics during normal stair climbing. Gait Posture.

[54] Mundermann A., Dyrby C.O., D’Lima D.D., Colwell C.W., Andriacchi T.P. (2008). In vivo knee loading characteristics during activities of daily living as measured by an instrumented total knee replacement. J. Orthop. Res..

[55] Taylor W.R., Schutz P., Bergmann G., List R., Postolka B., Hitz M., Dymke J., Damm P., Duda G., Gerber H. (2017). A comprehensive assessment of the musculoskeletal system: The CAMS-Knee data set. J. Biomech..

[56] Glitsch U., Heinrich K., Ellegast R.P. (2022). Estimation of Tibiofemoral and Patellofemoral Joint Forces during Squatting and Kneeling. Appl. Sci..

[57] Exoskeleton Report. https://exoskeletonreport.com/product/cray-x/.

[58] Kellis E., Baltzopoulos V. (1999). The effects of the antagonist muscle force on intersegmental loading during isokinetic efforts of the knee extensors. J. Biomech..

[59] Baltrusch S.J., van Dieen J.H., Bruijn S.M., Koopman A.S., van Bennekom C.A.M., Houdijk H. (2019). The effect of a passive trunk exoskeleton on metabolic costs during lifting and walking. Ergonomics.

[60] Park J.H., Lee Y., Madinei S., Kim S., Nussbaum M.A., Srinivasan D. (2022). Effects of Back-Support Exoskeleton Use on Lower Limb Joint Kinematics and Kinetics during Level Walking. Ann. Biomed. Eng..

[61] Alabdulkarim S., Kim S., Nussbaum M.A. (2019). Effects of exoskeleton design and precision requirements on physical demands and quality in a simulated overhead drilling task. Appl. Ergon..

